# Determining the optimal interval for imaging surveillance of ascending aortic aneurysms

**DOI:** 10.1007/s12471-021-01564-9

**Published:** 2021-04-13

**Authors:** B. P. Adriaans, M. J. F. G. Ramaekers, S. Heuts, H. J. G. M. Crijns, S. C. A. M. Bekkers, J. J. M. Westenberg, H. J. Lamb, J. E. Wildberger, S. Schalla

**Affiliations:** 1 Department of Cardiology, Maastricht University Medical Centre (MUMC+), Maastricht, The Netherlands; 2 Department of Radiology and Nuclear Medicine, Maastricht University Medical Centre (MUMC+), Maastricht, The Netherlands; 3 Cardiovascular Research Institute Maastricht (CARIM), Maastricht, The Netherlands; 4 Department of Radiology, Leiden University Medical Centre (LUMC), Leiden, The Netherlands; 5 Department of Cardiothoracic Surgery, Maastricht University Medical Centre (MUMC+), Maastricht, The Netherlands

**Keywords:** Aorta, Aortic aneurysm, Thoracic aortic aneurysm, Follow-up, Dissection

## Abstract

**Background:**

Cardiovascular guidelines recommend (bi-)annual computed tomography (CT) or magnetic resonance imaging (MRI) for surveillance of the diameter of thoracic aortic aneurysms (TAAs). However, no previous study has demonstrated the necessity for this approach. The current study aims to provide patient-specific intervals for imaging follow-up of non-syndromic TAAs.

**Methods:**

A total of 332 patients with non-syndromic ascending aortic aneurysms were followed over a median period of 6.7 years. Diameters were assessed using all available imaging techniques (echocardiography, CT and MRI). Growth rates were calculated from the differences between the first and last examinations. The diagnostic accuracy of follow-up protocols was calculated as the percentage of subjects requiring pre-emptive surgery in whom timely identification would have occurred.

**Results:**

The mean growth rate in our population was 0.2 ± 0.4 mm/year. The highest recorded growth rate was 2.0 mm/year, while 40.6% of patients showed no diameter expansion during follow-up. Females exhibited significantly higher growth rates than men (0.3 ± 0.5 vs 0.2 ± 0.4 mm/year, *p* = 0.007). Conversely, a bicuspid aortic valve was not associated with more rapid aortic growth. The optimal imaging protocol comprises triennial imaging of aneurysms 40–49 mm in diameter and yearly imaging of those measuring 50–54 mm. This strategy is as accurate as annual follow-up, but reduces the number of imaging examinations by 29.9%.

**Conclusions:**

In our population of patients with non-syndromic TAAs, we found aneurysm growth rates to be lower than those previously reported. Yearly imaging does not lead to changes in the management of small aneurysms. Thus, lower imaging frequencies might be a good alternative approach.

## What’s new?

- Nearly half of all ascending aortic aneurysms do not grow over time.
- Female sex and aortic regurgitation predispose to more rapid aortic growth.
- Triennial imaging follow-up suffices for ascending aortic aneurysms with diameters between 40 and 49 mm.

## Introduction

Acute type A aortic dissection (ATAAD) is a medical emergency that requires immediate surgery to avert fatal outcome. Dissection is commonly precipitated by aneurysm formation, and the risk for ATAAD is known to correlate with the maximal aneurysm diameter [1]. This risk is less than 1% per year in mildly dilated aortas but increases to 6.9% once the diameter reaches 60 mm [2–5]. To prevent dissection and rupture, prevailing guidelines recommend preventive extirpation of ascending aortic aneurysms ≥ 55 mm (or ≥ 50 mm in high-risk patients) [6–11]. Patients with smaller aneurysms require serial imaging until the threshold for intervention is reached.

Although indication criteria for prophylactic surgery rely on maximal diameter measurements, the optimal interval for the follow-up of thoracic aortic aneurysms (TAAs) has not been determined. If any, current guidelines propose surveillance protocols that involve (bi)annual computed tomography (CT) or magnetic resonance imaging (MRI) [6, 7]. However, these proposals are insufficiently backed up by data demonstrating the necessity for such an approach. The current retrospective longitudinal cohort study aims to (1) acquire growth rates of TAAs in the context of aortic valve morphology, (2) identify risk factors that predict the need for pre-emptive aortic repair, and (3) provide patient-specific follow-up intervals.

## Methods

### Study population

We reviewed the clinical database of the Maastricht University Medical Centre (MUMC+) for patients who were referred for TAA evaluation between January 1999 and August 2019. Patients with maximal ascending aortic diameters ≥ 40 mm at baseline who underwent multiple imaging examinations during follow-up were included. Exclusion criteria comprised prior aortic or valvular surgery and a diagnosis or suspicion of hereditary connective tissue disease. The study protocol was reviewed and approved by the institutional medical ethics committee, who waived the need for informed consent due to the study’s retrospective nature.

### Data collection and image analysis

Data collection and image assessment were performed by two readers (B.A., M.R.). Baseline characteristics and medical history were drawn from electronic patient records. Valve morphology and baseline valvular function were determined using transthoracic echocardiography (TTE) and classified according to current guidelines [12, 13].

All TTE, CT angiography, and MRI procedures performed for evaluation of TAA dimensions were assessed to study aortic diameters. Echocardiographic diameters were measured in parasternal long-axis views at end-diastole, using the leading edge-to-leading edge technique. CT and MRI were typically performed using prospective ECG gating with end-diastolic triggering, while measurements were acquired from inner edge to inner edge. For all modalities, care was taken to perform measurements perpendicular to the long axis of the aorta. Mean aneurysm growth rates were calculated by dividing the diameter difference between the first and last imaging examinations by the time interval between the two studies. The primary endpoint was reached when a patient met the indication criterion for pre-emptive surgery. This threshold was set at ≥ 55 mm (tricuspid aortic valve (TAV) and uncomplicated bicuspid aortic valve (BAV)) or ≥ 50 mm (BAV with concomitant risk factors (arterial hypertension, aortic coarctation, family history of dissection and/or expansion rate ≥ 3 mm/year)) [6, 8].

### Statistical analysis

Statistical analyses were performed using SPSS version 24 (IBM SPSS Statistics, Armonk, NY, USA). Continuous variables are presented as mean ± standard deviation (SD) or median and interquartile range (IQR). The Student *t*-test and Mann-Whitney U test were used for comparison between groups. Categorical variables are given as frequencies and percentages, and were compared using the chi-square test. Logistic regression was performed to identify predictors for reaching the endpoint of prophylactic surgery. For the patients who reached this endpoint, it was calculated—based on mean growth rates—whether a 2- or 3‑yearly imaging strategy would have delayed surgery compared to an annual surveillance approach. The diagnostic accuracy of various follow-up protocols was calculated as the percentage of subjects in whom timely identification would have occurred.

## Results

### Baseline characteristics

In total, 332 TAA patients (25.3% female) were included (Tab. 1). Median age was 64.2 years (range 19–86 years). Fifty-seven patients (17.2%) had BAV. Compared to those with BAV, patients with TAV were older (65.9 vs 52.4 years, *p* < 0.001) and more frequently suffered from hypertension (76.4% vs 43.9%, *p* < 0.001) and hyperlipidaemia (44.0% vs 17.5%, *p* < 0.001). Patients with BAV more often had moderate or severe aortic stenosis at baseline (28.1% vs 6.9%, *p* < 0.001).

**Table 1:** Baseline characteristics of the study population

	TAV (*n* = 275)	BAV (*n* = 57)	*p*-value
Age (years)	65.9 (58.4–71.7)	52.4 (42.4–59.8)	*<0.001*
Sex (female)	65 (23.6%)	19 (33.3%)	0.125
Height (cm)	174.8 ± 8.6	177.2 ± 9.6	0.07
Weight (kg)	81.8 ± 14.8	83.6 ± 14.8	0.39
*BMI (kg/m*^*2*^*)*	26.6 ± 3.9	26.6 ± 4.1	0.94
*BSA (m*^*2*^*)*	2.0 ± 0.2	2.0 ± 0.2	0.19
Hypertension	210 (76.4%)	25 (43.9%)	*<0.001*
Dyslipidaemia	121 (44.0%)	10 (17.5%)	*<0.001*
Diabetes	15 (5.5%)	3 (5.3%)	0.95
*AS* *≥* *moderate*	19 (6.9%)	16 (28.1%)	*<0.001*
*AR* *≥* *moderate*	27 (9.8%)	9 (15.8%)	0.19
CABG	25 (9.1%)	1 (1.8%)	0.06
CVA/TIA	35 (12.7%)	6 (10.5%)	0.65
Malignancy	33 (12.0%)	6 (10.5%)	0.75
Haemodialysis	2 (0.7%)	0	0.52

Values are median (interquartile range), number (percentage) or mean ± SD

*BMI* body mass index, *BSA* body surface area, *AS* aortic stenosis, *AR* aortic regurgitation, *CABG* coronary artery bypass graft, *CVA* cerebrovascular accident, *TIA* transient ischaemic attack,* SD* standard deviation

Median follow-up of the entire cohort was 6.7 years (IQR 4.5–10.2 years). During the cumulative follow-up of 2545.4 years, the included patients underwent 2233 imaging examinations (TTE *n* = 1750, CT *n* = 378, and MRI *n* = 105). Mean time between two consecutive measurements was 1.3 ± 0.7 years. Of the patients who died during follow-up (*n* = 23), none died of thoracic aortic disease. Two patients suffered dissection and underwent emergency surgery; both had TAV and diameters < 55 mm at last follow-up contact (both < 1 year before dissection onset).

### Aneurysm growth

**Fig. 1:**
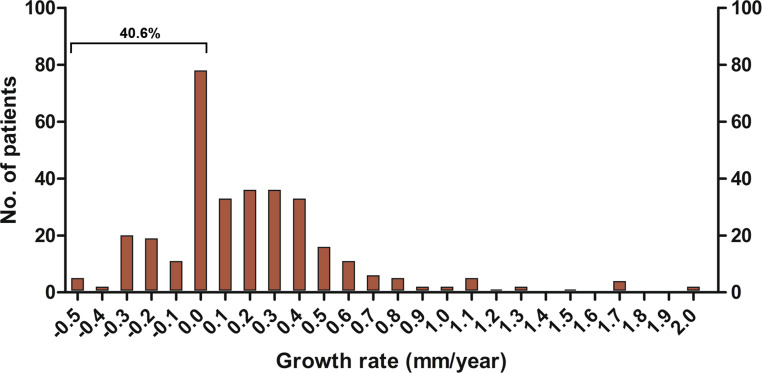
Histogram showing the distribution of mean growth rates among patients with thoracic aortic aneurysms. As depicted, the majority of aneurysms remain stable or grow only minimally over time

**Fig. 2:**
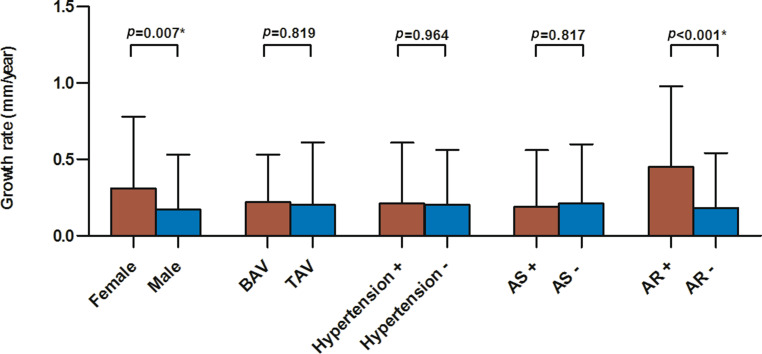
Bar charts showing mean growth rates of ascending aortic aneurysms as a function of sex, valve type, hypertension, and valvular function. Female sex and presence of more than moderate aortic regurgitation (*AR*) were associated with higher thoracic aortic aneurysm growth rates. *AS* aortic stenosis, *BAV* bicuspid aortic valve, *TAV* tricuspid aortic valve. *Statistically significant

The growth rates of the entire population were 0.14 mm/year (median) and 0.2 ± 0.4 mm/year (mean and SD). The largest observed growth rate was 2.0 mm/year, while 40.6% of patients showed no diameter increase between baseline and last follow-up (Fig. 1). Mean expansion rates were comparable between TAV and BAV (0.2 ± 0.4 mm/year vs 0.2 ± 0.3 mm/year, *p* = 0.819) (Fig. 2). Women had greater expansion rates than men (0.3 ± 0.5 vs 0.2 ± 0.4 mm/year, *p* = 0.007). At multivariable regression analysis, female sex and presence of at least moderate aortic regurgitation (AR) at baseline were shown to be independent predictors for more rapid aneurysm expansion (Tab. 2). In contrast, the presence of a BAV was not associated with higher growth rates.

**Table 2:** Univariable and multivariable regression analysis showing predictors for aortic growth

	Univariable		Multivariable	
	B	*p*-value	B	*p*-value
Age (years)	−0.003	0.108		
Sex (female)	0.134	* 0.007*	2.142	* 0.033*
Hypertension	0.002	0.964		
Dyslipidaemia	0.030	0.492		
Valve morphology (tricuspid)	0.013	0.819		
AS ≥ moderate	−0.016	0.817		
AR ≥ moderate	0.270	*<0.001*	3.987	*<0.001*
Baseline diameter (mm)	−0.011	0.118		

*AS* aortic stenosis,* AR* aortic regurgitation

### Surgical repair

During follow-up, 24 patients (7.2%) reached the indication criterion for pre-emptive surgery. Among those were 9 BAV patients, who all had concomitant risk factors and were referred for aneurysmectomy at a diameter of ≥ 50 mm. Logistic regression revealed baseline diameter, growth rate, and the predetermined interventional threshold to be independent predictors of reaching the primary endpoint (Tab. 3).

**Table 3:** Univariable and multivariable logistic regression analysis showing predictors for reaching the threshold for prophylactic aortic repair

	Univariable		Multivariable	
	B	*p*-value	B	*p*-value
Age (years)	−0.031	* 0.046*	0.024	0.578
Sex (male)	0.623	0.159		
Hypertension	0.003	0.996		
Dyslipidaemia	−0.089	0.839		
AS ≥ moderate	0.891	0.098		
AR ≥ moderate	1.609	* 0.001*	2.432	0.080
Surgical threshold (≥55 mm)	−1.179	* 0.009*	−5.610	* 0.004*
Baseline diameter (mm)	0.302	*<0.001*	1.130	*<0.001*
Growth rate (mm/year)	3.992	*<0.001*	11.299	*<0.001*

*AS* aortic stenosis, *AR* aortic regurgitation

### Optimal imaging interval

Various imaging protocols were tested for their accuracy in timely identification of the necessity for pre-emptive aortic repair (Tab. 4 and 5). Lowering imaging frequencies to less than once annually causes delay to surgery in patients in whom the current diameter is within 5 mm of the surgical threshold. However, imaging intervals can be prolonged safely to once every 3 years, as long as the diameter is between 40 and 44 mm (BAV with risk factors) or 40–49 mm (uncomplicated BAV and TAV). The implementation of such strategies would have reduced the number of imaging examinations in the study population by 16.0% and 29.9%, respectively.

**Table 4:** Diagnostic accuracy of various imaging strategies. *Surgical threshold: ≥* *55* *mm*

	Current diameter			Accuracy (%)	Reduction of imaging studies^a^ (%)
	40–44 mm	45–49 mm	50–54 mm		
Protocol 1	Two-yearly	Two-yearly	Annually	100	17.8
Protocol 2	Two-yearly	Two-yearly	Two-yearly	53.3	43.4
Protocol 3	Three-yearly	Two-yearly	Annually	100	20.6
Protocol 4	Three-yearly	Three-yearly	Annually	100	29.9

^a^As compared with a once-yearly strategy

**Table 5:** Diagnostic accuracy of various imaging strategies. *Surgical threshold: ≥* *50* *mm*

	Current diameter		Accuracy (%)	Reduction of imaging studies^a^ (%)
	40–44 mm	45–49 mm		
Protocol 1	Two-yearly	Annually	100	8.0
Protocol 2	Two-yearly	Two-yearly	33.3	44.4
Protocol 3	Three-yearly	Annually	100	16.0

^a^As compared with a once-yearly strategy

## Discussion

Cardiovascular guidelines provide comprehensive recommendations on which patients should be selected for preventive aortic repair. However, the management of asymptomatic non-syndromic TAAs with diameters below interventional cut-offs is less well described. Our main conclusions are: (1) the majority of TAAs remain stable or grow minimally over time, (2) the need for prophylactic surgery is determined by baseline diameter, growth rate, and the predetermined interventional threshold, and (3) 3-yearly imaging follow-up suffices for any aneurysm with a diameter > 5 mm below the surgical indication criterion.

### Aortic growth rates

Thus far, studies that sought to investigate aneurysm growth rates have yielded divergent results. Our findings concur with those of recent investigations, which have shown that ascending aortic aneurysms expand at slightly higher rates than the normal-sized aorta (i.e. 0.2–0.4 mm/year vs 0.1 mm/year) [14–17]. As such, average TAA growth rates are markedly lower than previously assumed [18]. Pioneer work from the Yale group, whose studies form the basis for many recommendations within current guidelines, demonstrated an average enlargement of 0.7–1.2 mm/year [19]. Using serial CT, Hirose et al. even documented growth rates as high as 4.2 mm/year [20]. However, these early studies included a substantial number of patients with connective tissue disease and chronic dissections, who were followed over a relatively short period (i.e. 6–19 months). Since short follow-up durations can cause small diameter changes and measurement inaccuracies to become translated into disproportionally high annual growth rates, it is conceivable that the expansion rates reported in these studies are somewhat overestimated. In comparison, a growth rate of ≥ 0.7 mm/year was observed in only 8.1% of our patient population.

The current study provides more insight into the individual character of TAA progression. Based on reported mean expansion rates, it has long been thought that all aneurysms grow over time [8, 19]. However, our results reflect that nearly half of non-syndromic TAAs remain stable in size—even at long-term follow-up. In contrast, a small portion of patients exhibit remarkably high growth rates (up to eight times the mean) and are at risk for reaching the diameter threshold for preventive surgery. Moderate or severe AR at baseline was shown to be the most important risk predictor for rapid growth. The pathophysiological mechanism behind this observation can be regarded as a vicious circle, in which AR causes increase of left ventricular stroke volumes and elevated aortic wall stress, and further aortic dilatation leads to aggravation of AR. Importantly, we have shown that presence of BAV does not confer risk for more rapid aneurysm dilatation. This, and the observation that BAV is not associated with a higher risk of acute aortic events, may question the lower interventional thresholds that apply to this patient group [3, 21].

Our data confirm that female sex is associated with more rapid aneurysm growth [22]. Moreover, outcome studies have demonstrated that female TAA patients have a three-fold higher risk of experiencing dissection, and a 40% increase in risk of mortality [19, 23]. Although the magnitude of sex-based growth differences does not merit a more frequent imaging strategy in women, it prompts deliberation as to why the natural history of aneurysms differs between genders. One theory explains the worse outcome in women by their higher vessel wall concentrations of matrix metalloproteinases (MMPs)—enzymes involved in the degradation of extracellular matrix (ECM) content. Interestingly, aneurysms with more pronounced MMP‑2 and MMP‑9 expression show more extensive ECM remodelling and decreased compliance [24]. Future immunohistochemical and biomechanical studies could evince a causal relation between gender-associated structural differences and the more aggressive TAA behaviour in women.

### Implications for imaging

Current guidelines prescribe referral for preventive surgery based on absolute diameter cut-offs. In addition, the ACC/AHA advocates elective operation of patients who exhibit growth rates > 5 mm/year. Assessment of these indications requires adequate imaging surveillance, yet only two out of five available guidelines provide appropriateness criteria to this end. Based on the consensus opinion of experts, these recommend yearly or semi-annual CT or MRI [6, 7]. Although others cautiously suggest lowering imaging frequencies in the case of stable disease, no previous study systematically investigated the impact of different follow-up protocols. The results of the current study reveal that yearly imaging carries no therapeutic consequences for patients with small aneurysms. On the contrary, it can add unjustifiable costs to health care and unnecessary exposure to radiation and contrast agent administration. Here, we propose a surveillance strategy that consists of 3‑yearly imaging of patients with smaller aneurysms and yearly imaging of those who are in close proximity to their respective surgical thresholds. We conclude that this approach is as accurate as annual follow-up and reduces the number of requested imaging examinations by 29.9% (TAV) and 16.0% (BAV with risk factors). It is noteworthy that these percentages have been calculated using data of patients who met the endpoint of prophylactic surgery. The proposed strategy could have reduced imaging frequencies by up to two-thirds in patients with stable diameters.

Although current guidelines primarily depend on maximal diameter cut-offs, it is generally known that ATAAD can also occur in aortas that do not meet criteria for pre-emptive surgical repair [25]. Recent studies have investigated the predictive potential of aortic length and volume, and found that these may improve the timely identification of patients at risk [26, 27]. Furthermore, four-dimensional (4D) flow MRI is emerging as a clinically feasible imaging technique that can further improve selection of patients who may benefit from prophylactic surgery [28, 29]. Future prospective studies are now needed for these novel imaging markers to find their way into clinical guidelines.

### Limitations

Some limitations of the current study need to be addressed. First, our study population is not representative of patients with connective tissue diseases. These are reported to have higher aortic growth rates, and the proposed imaging intervals should not be extrapolated to this patient group [30]. Second, we have only looked into growth rates of ascending aortic aneurysms. Descending thoracic and abdominal aneurysms may expand more rapidly and merit more frequent surveillance imaging. Since we included only patients who underwent imaging at ≥ 2 different time points, we were not able to adequately evaluate the risk of adverse outcomes. Third, diameters were assessed using different imaging modalities. Although leading edge-to-leading edge (TTE) and inner edge-to-inner edge (CT and MRI) measurements closely agree, incongruity between methods may have contributed to inaccuracies in calculated growth rates [31]. Fourth, growth rates were calculated assuming a linear TAA growth pattern. According to Laplace’s law, wall tension is proportional to the vessel radius for a given blood pressure. Therefore, it has been hypothesised that growth rates become higher as the aorta progressively enlarges [32]. However, a previous study with standardised imaging intervals could not confirm this hypothesis and found that growth rates remained steady during follow-up [15]. Due to the various time points at which our patients were imaged, we were not able to investigate the relationship between aortic size and growth rate in the current study. Finally, this was a single-centre analysis. Future prospective multicentre studies are needed to confirm our results.

## Conclusions

Non-syndromic ascending aortic aneurysms grow at lower rates than previously assumed. Reduction of imaging frequency to once every 3 years seems reasonable for asymptomatic patients in whom the ascending aorta is only mildly dilated.
